# Factors influencing health-related quality of life among long-term care residents experiencing pain: a systematic review protocol

**DOI:** 10.1186/s13643-024-02459-7

**Published:** 2024-02-01

**Authors:** Shovana Shrestha, Greta Cummings, Jennifer Knopp-Sihota, Rashmi Devkota, Matthias Hoben

**Affiliations:** 1 Faculty of Nursing, College of Health Sciences, University of Alberta, Edmonton, Alberta Canada; 2 Faculty of Health Disciplines, Athabasca University, Edmonton, Alberta Canada; 3 School of Health Policy and Management, Faculty of Health, York University, Toronto, Ontario Canada

**Keywords:** Quality of life, HRQoL, Pain, Older adults, Elderly, Aged, Long-term care

## Abstract

**Background:**

Pain is highly burdensome, affecting over 30% of long-term care (LTC) residents. Pain significantly reduces residents’ health-related quality of life (HRQoL), limits their ability to perform activities of daily living (ADLs), restricts their social activities, and can lead to hopelessness, depression, and unnecessary healthcare costs. Although pain can generally be prevented or treated, eliminating pain may not always be possible, especially when residents have multiple chronic conditions. Therefore, improving the HRQoL of LTC residents with pain is a priority goal. Understanding factors influencing HRQoL of LTC residents with pain is imperative to designing and evaluating targeted interventions that complement pain management to improve residents’ HRQoL. However, these factors are poorly understood, and we lack syntheses of available research on this topic. This systematic review protocol outlines the methods to identify, synthesize, and evaluate the available evidence on these factors.

**Methods:**

This mixed methods review will follow the Preferred Reporting Items for Systematic Reviews and Meta-Analysis (PRISMA) guidelines. We will systematically search Medline, EMBASE, PsycINFO, CINAHL, Scopus, Cochrane Database of Systematic Reviews and ProQuest Dissertation and Thesis Global from database inception. We will include primary studies and systematically conducted reviews without restrictions to language, publication date, and study design. We will also include gray literature (dissertation and reports) and search relevant reviews and reference lists of all included studies. Two reviewers will independently screen articles, conduct quality appraisal, and extract data. We will synthesize results thematically and conduct meta-analyses if statistical pooling is possible. Residents and family/friend caregivers will assist with interpreting the findings.

**Discussion:**

This proposed systematic review will address an important knowledge gap related to the available evidence on factors influencing HRQoL of LTC residents with pain. Findings will be crucial for researchers, LTC administrators, and policy makers in uncovering research needs and in planning, developing, and evaluating strategies in addition to and complementary with pain management to help improve HRQoL among LTC residents with pain.

**Systematic review registration:**

PROSPERO CRD42023405425

**Supplementary Information:**

The online version contains supplementary material available at 10.1186/s13643-024-02459-7.

## Background

Growth in the number of older adults is a global phenomenon [1–3]. Along with the population’s increasing age, the numbers of older adults with multiple chronic illnesses [4–6] and complex care needs are also rising, substantially increasing the need for residential long-term care (LTC) [7, 8]. LTC provides accommodation to people requiring on-site access to supervised care around the clock, including professional health care services, personal care, and services such as meals, laundry, and housekeeping [9]. More than 50% of LTC residents are 85 years and older, 70% are female, 90% are cognitively impaired, and 80% need assistance with activities of daily living (ADLs) [10, 11]. Many LTC residents suffer from multiple chronic conditions (an average of six chronic conditions) [12], including arthritis, osteoporosis, depression, hypertension, and dementia [13–15]. Chronic conditions increase the risk for pain in LTC residents [12, 16, 17]—a common and burdensome condition [18, 19].

Pain is “an unpleasant sensory and emotional experience associated with, or resembling that of actual or potential tissue damage” [20] ^(p.2)^. Internationally, pain prevalence estimates among LTC residents vary widely between 31 and 70% [13, 21–23]. Given that pain can generally be prevented or treated, these rates are unacceptably high [18, 19]. Pain severely impacts residents’ health-related quality of life (HRQoL) [24]. It impairs residents’ mobility and independence [25, 26] and contributes to hopelessness, insomnia, loneliness, depression, poor social relationships, and unnecessary health care costs [24, 27, 28]. However, even when applying best practices in pain management [29–31], eliminating pain is not always possible, especially when residents have multiple comorbidities [12, 32, 33]. Therefore, for residents living with pain, in addition to treating their pain as best as possible, preserving their functional abilities, and supporting their best possible HRQoL is a priority goal [34, 35]. HRQoL reflects those aspects of QoL that directly or indirectly relate to an individual’s perception of the impact a health condition, illness, or treatment has on their life [36, 37]. Recognizing that many factors influence LTC residents’ HRQoL [38–40], we define factors for this review as any condition influencing the HRQoL of residents experiencing pain [41]. These conditions include (1) resident characteristics [40, 42, 43], (2) socio-economic aspects [39, 44, 45], and (3) LTC facility characteristics [46–48]. Studies suggest that younger residents [49], female [38, 50], married [49], and financially secure [51, 52] reported better QoL. Residents who are dependent on with ADLs [40, 46], who experience pain [24, 40], have anxiety/mood disorders [49] and depression [38, 46], and who are cognitively impaired [38, 40, 50] have reduced QoL. Evidence also suggests that residents can maintain high QoL despite co-morbid health conditions, including dementia [39, 53], pain [38, 54], and diminished physical and cognitive functioning [38, 54]. Internal personal resources, such as resilience [45], meaning or purpose in life [55], sense of coherence [56, 57], and religiosity/spirituality [40, 46] effectively helped residents to cope with health and life adversities (including pain). Similarly, residents who perceived better support from family/friends and LTC staff in terms of their availability, attachment, and quality of relationship had a better HRQoL [39, 56].

Past reviews mainly focused on factors influencing the QoL of LTC residents in general or those with dementia [40, 42, 43]. However, to the best of our knowledge, no literature synthesis is available on factors that explain variation in HRQoL among LTC residents with pain. Since the best possible HRQoL is a priority goal of care for all LTC residents, especially among those whose pain cannot be removed entirely, we must understand what helps residents with pain to achieve the best possible HRQoL [58, 59]. Identifying these factors is fundamental for planning and developing targeted interventions—in addition and complementary to best practices in pain management—to support LTC residents with pain in maintaining or improving HRQoL. This systematic review protocol details the methods we will use to identify factors that influence HRQoL among LTC residents with pain. We aim to identify, evaluate, and synthesize the available research evidence on the factors that are associated with HRQoL of LTC residents experiencing pain. Our research questions are: (1) what factors associated with HRQoL of LTC residents experiencing pain do studies report? (2) What is the magnitude, direction, and strength of evidence of each factor’s association with HRQoL of LTC residents experiencing pain?

## Methods/design

### Review design

We will conduct a systematic mixed methods synthesis of research [60]. This paper follows the Preferred Reporting Items for Systematic Reviews and Meta-Analysis Protocols (PRISMA-P) checklist (see Additional file 1) [61]. We will follow the procedures outlined in the Cochrane Handbook of Systematic Reviews [62] and Preferred Reporting Items for Systematic Reviews and Meta-Analyses (PRISMA) checklist [63] for review methods and reporting of results. The protocol for this systematic review has been registered with the International Prospective Register of Systematic Reviews (PROSPERO) with registration number CRD42023405425.

### Inclusion and exclusion criteria for study selection

#### Participants

We will include studies that examined LTC residents aged 65 years and older. The average or median age of participants must be at least 65 years. Studies where participants are referred to as aged/elderly/older individuals, seniors, or residents of a continuing care institution will also be included. Studies must have included LTC residents with pain, either acute (lasting less than 3 months) or chronic pain (lasting 3 months or more) [64] assessed using a standardized clinical assessment tools (based on resident self-reports, proxy-reports or clinical assessments/observations). No further limitations on participant characteristics will be applied. Study inclusion will not be limited based on participant sex/gender, race/ethnicity, or any other social identity or demographic feature.

#### Outcomes

We will include studies that assessed HRQoL as a primary or secondary outcome. In particular, we will include studies if they report the association of factor(s) with the HRQoL of LTC residents who live with pain. The revised version [65] of Wilson’s & Cleary’s health-related quality of life (HRQoL) model and the Consolidated Framework for Implementation Research (CFIR) developed by Damschroder et al. [66] will inform the factors. The Zubrtisky et al. [65] model suggests that resident characteristics (physiological health, emotional health, functional status, general health perceptions, beliefs, and behaviors) and the environment where residents live (service delivery system, structure) shape their HRQoL. While this model includes the environment as one of many factors that influence resident HRQoL, it does not explicitly operationalize this important factor. Therefore, in addition to this model, we will also use the CFIR which will help further operationalize the LTC organizational context (work environment) construct. The CFIR framework defines context or environment as the “set of circumstances or unique factors that surround a particular implementation effort” [66] ^(p.3)^. For this review, we will use the CFIR dimension called inner setting to operationalize the construct of organizational context. The inner setting includes structural characteristics, networks and communication, culture, climate, and readiness, which interact with each other to influence implementation [66]. Both frameworks (HRQoL and CFIR) will inform the identification of articles that report factors of interest, theme generation during data extraction and analysis, and interpretation of results. In line with the above theoretical frameworks, factors in this review may include (a) resident factors (physical and cognitive functioning, resilience, coping strategies, optimism, purpose in life); (b) socio-economic factors (social support, social engagement, economic resource); (c) LTC factors (LTC characteristics, care practices, policies, care model, care home organizational context such as leadership, staffing, resources, work culture, evaluation, communication).

#### Types of studies

We will include primary empirical studies and systematically conducted reviews (i.e., reviews that included comprehensive search strategy, reported inclusion/exclusion criteria, screening process, data extraction and analysis of the included studies) without any restrictions on the language, study design, and publication date. Our study team includes members who can assess the eligibility of studies published in English, French, German, and Nepali language. For studies in other languages, we will utilize our networks of colleagues to identify persons who might be able to help with assessing eligibility, or use Google Translate to assess eligibility. We will include gray literature (dissertations, theses, and reports) identified in the database searches, but we will not systematically search gray literature. In addition to the electronic database search, we will also search the reference lists of all the included studies and relevant reviews. We will exclude non-empirical and non-systematic review studies. We will include studies conducted in LTC also referred to as nursing homes, care homes, and residential aged care facilities [9, 15, 67–69] (detailed inclusion and exclusion criteria in Appendix 1).

### Search strategy

Assisted by a health sciences librarian (MK), we developed the search strategy (see Appendix 2 for search strategy created for various databases). Search terms were based on four major concepts and their synonyms: (1) older adults (seniors), (2) care location (LTC, nursing homes), (3) pain, and (4) quality of life. We searched the following databases: Medline, Embase, PsycINFO, CINAHL, Scopus, Cochrane Database of Systematic Reviews and ProQuest Dissertations and Theses Global from database inception till date. We will retrieve all studies without limiting language, study design, and publication date. Further we will search the reference lists of all the studies included and relevant reviews.

### Data management

Records will be managed, using COVIDENCE systematic review software [70]. Reviewers will receive training on using COVIDENCE prior to screening articles. We will use COVIDENCE for de-duplication, title/abstract screening, full-text screening, and monitoring and reporting of reviewer agreement.

### Study screening

Two reviewers (SS and RD) will screen titles and abstracts of 100 papers independently, followed by a calibration meeting. If the first round demonstrates discrepancies in decisions related to publication type, setting, population, and study focus, we will do additional calibration rounds and reconcile decisions with a third reviewer (MH). Using a similar approach, we will screen the remaining titles and abstracts in batches. We will obtain full texts of all included studies and for those with inadequate information in the title/abstract screening to decide on inclusion. We will screen full texts using the same approach as for the title/abstract screening. Two reviewers (SS and RD) will independently screen reference lists of all included studies and reviews for any additional relevant studies. We will document reasons for each article’s exclusion and the selection process using a PRISMA-ScR flow diagram [61].

### Data extraction

We will construct a Google form to directly abstract relevant data. To test useability of the data extraction form (Google form), we will pilot-test it using five randomly selected studies [71]. Two reviewers (SS and RD) will independently extract data from an initial 10% of the studies, followed by a consensus meeting to calibrate extraction. Reviewers will discuss discrepancies and reach a consensus before extracting data from the remaining studies. One reviewer (SS) will extract data for the remaining studies, and another (RD) will double-check the extracted data for accuracy. Data extraction will include (1) surname of the first author, (2) year of publication, (3) the title of study, (4) country of study, (5) study aim(s) or purpose(s), (6) study design, (7) setting and sample, (8) method(s) of data collection, (9) tools and measures used to assess HRQoL and pain (for quantitative studies) or phenomena assessed (qualitative studies), (10) definition of pain, (11) statistical analyses methods used, and (12) main study findings: factors influencing HRQoL of LTC residents with pain.

### Risk of bias

Two reviewers will independently assess the methodological quality of all included studies. However, we will not exclude studies based on their quality. Reviewers will meet to reconcile any discrepancies. We will assess the risk of bias using validated and reliable tools depending on the study design of included studies. We will employ the validated and reliable AMSTAR 2 (Assessing the Methodological Quality of Systematic Reviews 2) tool [72, 73] for systematically conducted reviews, Quality Assessment Tool for Quantitative Studies (QATQS) [74–76] for quantitative studies (e.g., randomized and non-randomized, cohort and case studies); the Appraisal Tool for Cross-sectional Studies (AXIS) [77] for cross-sectional studies; Critical Appraisal Skills Program (CASP) Qualitative Research Checklist [78] for qualitative studies; and the Mixed Methods Appraisal Tool (MMAT) [79] for mixed-method studies.

We will use the scoring method developed by de Vet et al. [80] to obtain an overall quality rating for each study. de Vet et al.’s method uses the total number of checklist items that are applicable to a study as the denominator and the items that study meet as the numerator. Therefore, for each checklist and assessment, we determine the percentage of items that each study meet. Although each quality appraisal tool/checklist varies in the number of items and criteria applied, they still have in common that ideally a study should meet all required criteria and that meeting a higher number of criteria is considered better study quality. Therefore, de Vet et al.’s method is a form of standardization across checklists. Scoring will provide us with an idea about the study’s methodological quality, which is crucial in informing decision-making based on the quality of evidence [80]. We will calculate the ratio of the obtained score to the maximum possible score, which varies with the checklist used and the number of items in the checklists that apply to the respective study. The score ranges from 0 to 1. Similar to previous studies [81, 82], we will categorize the quality of studies as weak (≤ 0.50), low moderate (0.51–0.66), high moderate (0.67–0.79), or strong (≥ 0.80). In addition, we will also conduct a quality assessment on the item level for each study based on a particular quality appraisal tool.

### Data analyses and synthesis

Using tables and figures, we will descriptively present the number and proportion of studies representing each category: number of studies, year of publication, countries of origin, study design, study settings, participants’ details, and quality of studies. We will conduct thematic analysis of all studies. For thematic analysis [83], two independent reviewers will familiarize and inductively code data, look for similarities and differences between the codes, and then group them into similar themes or categories. Acute (pain lasting less than 3 months) and chronic pain (pain lasting 3 months or more) [64] are different. Further, pain may be evaluated using self, proxy, or observation-based assessment tools. Accordingly, factors affecting residents’ HRQoL may be different in residents with acute and chronic pain and in residents assessed using self, proxy, or observation-based pain assessment [28]. Similarly, prior studies have shown that QoL in LTC residents may vary by sex/gender [38], race/ethnicity [49, 84], study country of origin [85], and LTC organizational context [86, 87]. Therefore, we will conduct sub-group analysis to report factors associated with HRQoL by type of pain (acute/chronic), pain assessment methods (self-report, proxy-report, observation), sex/gender, race/ethnicity, study country of origin, and LTC organizational context if we identify enough studies that allow stratification. The HRQoL [65] and CFIR [66] framework will help to categorize themes or factors influencing HRQoL of LTC residents with pain. We will resolve any discrepancies in the process by consensus. After thematic analysis, we will provide a narrative synthesis of results—a textual approach to understanding and reporting findings [88]. We will report the number of studies reporting each of the factors identified.

For qualitative results, we will conduct a content analysis [88]. In this process, we will identify key themes and assess if these key themes vary (if they are similar or different) across studies. We will then categorize themes into either resident, socio-economic, or LTC-related factors as informed by the HRQoL [65] and CFIR [66] framework. For quantitative findings, we will summarize the available quantitative evidence such as effects sizes of correlations, regression parameters, relative risks, or odd ratios. We will report the range of scores, frequency and proportion of studies reporting statistically significant positive associations, statistically significant negative associations, and statistically non-significant associations for association of factors with our study outcome based on vote counting.

If there are a sufficient number of quantitative studies reporting similar designs, settings, and outcomes, then we will statistically pool results of these studies using random effects meta-analysis [62]. Three or more studies are required to estimate measures of heterogeneity in addition to estimating pooled effects for random-effects meta-analysis [89]. For this study, we will conduct statistical pooling, if three or more studies (1) report similar factors associated with residents’ HRQoL, (2) measure HRQoL and pain in a comparable way (i.e., using a comparable measurement tool), (3) report the same resident outcome (HRQoL of LTC residents with pain), and (4) report the same type of statistical outcome. We will calculate the extent of heterogeneity using the *I*^2^ [90, 91] and *H*^2^ statistic [92] including their 95% confidence intervals [90, 91]. We will use random effects models using the statistical software SPSS (Statistical Package for Social Sciences), version 22 [93]. Random effects models are considered better than fixed-effects models in case of heterogeneity and a small number of studies [94, 95]. We will also assess if study protocols are available for the included studies (particularly randomized controlled trials) and if they were published before recruitment of patients. We will assess publication bias using funnel plots if we are able to include more than ten comparable studies because publication bias is difficult to evaluate among ten or fewer studies due to lack of power [96]. If the studies included in the review are too small in number and are heterogeneous then we will only conduct thematic analysis of the review findings as described above. We will assess the overall quality of the body of evidence using the Grading of Recommendation Assessment, Development and Evaluation guidelines (GRADE) [97]. According to GRADE, quality will be categorized as very low (any estimate of effect is very uncertain), low (further research is very likely to have a significant impact on our confidence in the estimate of effect and is likely to change the estimate), moderate (further research is likely to have an important impact on our confidence in the estimate of effect and may change the estimate), and high (further research is very unlikely to change our confidence in the estimate of effect) [97].

### Stakeholder consultation

Consultation enhances a review’s relevance by integrating stakeholders’ lived experience, expertise, and perspective [98]. We plan to consult with stakeholders after synthesizing the review findings. Stakeholders will contribute meaningfully in interpreting findings in terms of whether associations reflect their experiences and reality [98]. Using our existing stakeholder networks, we will include four stakeholders as an advisory group: (a) two LTC residents with pain who can verbally express their opinion and (b) two family/friend caregivers. To recruit stakeholders, first, we will purposively select a LTC from our existing network. Second, we will contact care home administrator via email or phone and request them to identify potential stakeholders and get permission for us to contact them directly. We will then approach selected stakeholders, explain the purpose of their involvement, and obtain their verbal consent. We will conduct a semi-structured interview using recorded virtual focus group discussion (lasting about an hour). The analysis will involve theme generation, and we will integrate findings in the result section separately in a narrative form.

## Discussion

Given that improving HRQoL is a priority goal in LTC residents living with pain and that there is a lack of synthesis in this topic, we aim to identify, synthesize, and evaluate available evidence on factors that are associated with HRQoL of LTC residents with pain. Identifying factors, particularly those that are modifiable and those that are positively associated with residents’ HRQoL will help us plan, develop, and evaluate tailored strategies to improve residents’ HRQoL. Further, we will be able to identify research gaps on the topic, which can inform future studies. Stakeholders’ involvement will further add credibility to the findings as they will help interpret the findings in terms of whether the associations observed reflect their experiences and reality. The findings will be crucial for researchers, LTC administrators and policymakers.

### Supplementary Information

**Additional file 1.** PRISMA-P 2015 Checklist.

## Data Availability

We will include all data generated or analyzed in the published systematic review article. Upon request, other resources can be made available.

## Appendix 1

**Table 1:** Inclusion and exclusion criteria

**Criteria**	**Inclusion criteria**	**Exclusion criteria**
**Study design**	Empirical studies, regardless of study designs (qualitative, quantitative, and mixed) that meet the review objective. Quantitative studies • Randomized trials • Non-randomized trials • On-group pre-post studies • Cohort studies • Case-control studies • Cross-sectional studies • Time-series analyses Qualitative studies • Qualitative interviews • Focus groups • Ethnographic observations • Qualitative case studies Mixed methods studies Systematically conducted reviews • Meta-analyses • Systematic reviews • Realist reviews • Integrative reviews • Scoping reviews • Narrative reviews	Non-empirical studies (editorials, opinion texts, theoretical discussions, commentaries, case reports, newspaper, historical articles, lecture notes, presentations, personal narratives) Non-systematically conducted reviews. • Meta-analyses • Systematic reviews • Realist reviews • Integrative reviews • Scoping reviews • Narrative reviews
**Setting**	LTC homes, also referred to as nursing homes, care homes and aged care facilities	Hospital, community, home care (private homes), assisted living/supportive housing, retirement homes, and equivalent settings
**Study Population**	Studies in which the average or the median age of residents is ≥ 65 years, or studies where participants are referred to as aged/elderly/older individuals, seniors, or residents. Studies that included older and younger adults will be included if results for people aged ≥ 65 years are reported separately. Residents must have pain, either acute or chronic assessed using a standardized clinical assessment tool We will focus on both acute (sudden onset) or chronic (pain lasting 3 months or more) [64] in this study	Studies that do not include participants aged ≥ 65 years. Studies that include participants aged 65 years or older but that do not report results of people aged ≥ 65 years separately
**Study Focus**	Studies including HRQoL as primary, or secondary outcomes assessed using a formal HRQoL assessment tool. Studies reporting statistically significant positive association, negative association, or non-significant association between factors and HRQoL for LTC residents with pain. Factors may include: (a) resident factors: physical and cognitive functioning, resilience, coping strategies, optimism, purpose in life). (b) socio-economic factors: social support, social engagement, economic resource) [38, 39, 44, 45, 99, 100]; and c) LTC factors: characteristics, care practices, policies, care model, care home organizational context such as leadership, staffing, resources, work culture, evaluation, communication, etc.	Intervention studies that exclusively report the effectiveness of pain treatment approaches including pharmacological (i.e., analgesic, and non-analgesic drugs) and non-pharmacological approaches (e.g., music therapy, tai chi, acupuncture, etc.)

## Appendix 2

### Search strategies created for various database

**Database**: Ovid MEDLINE(R) ALL < 1946 to August 4, 2022 > **(Total searches: 715)**

1. (longterm care or long term care or LTC).mp.
2. exp Geriatrics/ or Aged/ or Health Services for the Aged/ or Senior Centers/ or (elders or elderly or geriatric* or old age or (seniors not “high school”) or (older adj3 (adult*or person* or people or man or men or woman or women)) or centenarian* or nonagenarian* or octogenarian* or septuagenarian* or sexagenarian* or dottering or decrepit or tottering or overaged or “oldest old”).mp.
3. 1 and 2
4. exp Nursing Homes/ or exp Homes for the Aged/ or exp Rehabilitation Centers/ or exp Skilled Nursing Facilities/ or (nursing home* or extended care* or care home*).mp. or ((senior* or continuing care or disabled or old age or geriatric* or elder care* or rehabilitat* or long term care) adj2 (lodge* or facility* or home* or residence* or centre* or center*)).mp.
5. 3 or 4
6. exp pain/
7. (pain* or discomfort* or soreness or ache* or aching or hurt*).mp.
8. 6 or 7
9. “Quality of Life”/
10. (“quality or life” or QoL or HRQoL or HQoL).mp.
11. (“life satisfaction” or “life-satisfaction”).mp.
12. (“wellbeing” or “well-being”).mp.
13. 9 or 10 or 11 or 12
14. 5 and 8 and 13

**Database**: EMBASE < 1974 to August 4, 2022 > **(Total searches: 2054)**

1. (longterm care or long term care or LTC).mp.
2. exp geriatrics/ or aged/ or aged hospital patient/ or exp elderly care/ or frail elderly/ or gerontology/ or institutionalized elderly/ or very elderly/ or (“aging in place” or elders or elderly or geriatric* or gerodontic* or old age or (seniors not “high school”) or (older adj3 (adult* or person* or people or man or men or woman or women)) or centenarian* or nonagenarian* or octogenarian* or septuagenarian* or sexagenarian* or dottering or decrepit or tottering or overaged or “oldest old”).mp.
3. 1 and 2
4. exp nursing home/ or exp home for the aged/ or exp rehabilitation center/ or institutionalized elderly/ or (nursing home* or extended care* or care home*).mp. or ((convalescen* or senior* or continuing care or disabled or old age or geriatric* or elder care* or rehabilitat* or long term care) adj2 (lodge* or facility* or home* or residence* or centre* or center*)).mp.
5. 3 or 4
6. exp pain/
7. (pain* or discomfort* or soreness or ache* or aching or hurt*).mp.
8. 6 or 7
9. “Quality of Life”/
10. (“quality or life” or QoL or HRQoL or HQoL).mp.
11. (“life satisfaction” or “life-satisfaction”).mp.
12. (“wellbeing” or “well-being”).mp.
13. 9 or 10 or 11 or 12
14. 5 and 8 and 13

**Database**: PsychInfo < 1974 to August 4, 2022 > **(Total searches: 302)**

1. (longterm care or long term care or LTC).mp.
2. geriatrics/ or exp “aged (attitudes toward)”/ or exp aging/ or geriatric assessment/ or geriatric psychotherapy/ or gerontology/ or exp geropsychology/ or late life depression/ or exp elder care/ or elder abuse/ or (elders or elderly or geriatric* or old age or oldest age* or (seniors not “high school”) or (older adj3 (adult* or people or person or persons or man or men or woman or women)) or oldest patient* or “old old” or “very old” or centenarian* or nonagenarian* or octogenarian* or septuagenarian* or sexagenarian* or dottering or decrepit or tottering or “late* life” or overaged or “oldest old”).ti,ab,jn,jx,mh,sh.
3. 1 and 2
4. exp Nursing Homes/
5. exp Residential Care Institutions/
6. exp Rehabilitation Centers/
7. institutionalized elderly.mp.
8. (nursing home* or extended care* or care home*).mp.
9. ((senior* or continuing care or disabled or old age or geriatric* or elder care* or rehabilitat* or long term care) adj2 (lodge* or facility* or home* or residence* or centre* or center*)).mp.
10. 4 or 5 or 6 or 7 or 8 or 9
11. exp Pain/
12. (pain* or discomfort* or soreness or ache* or aching or hurt*).mp.
13. 11 or 12
14. “Quality of Life”/
15. (“quality or life” or QoL or HRQoL or HQoL).mp.
16. (“life satisfaction” or “life-satisfaction”).mp.
17. (“wellbeing” or “well-being”).mp.
18. 14 or 15 or 16 or 17
19. 3 or 10
20. 13 and 18 and 19

**Database**: CINAHL < 1937 to August 4, 2022 > (**Total searches: 1198)**

1. (MH “Long Term Care”) OR “(longterm care or long term care or LTC).mp.”
2. (MH “Geriatrics”) or (MH “Aged, Hospitalized”) or (MH “Aged+”) or (MH “Senior Centers”) or (MH “Gerontologic Care”) or (MH “Geriatricians”) OR (MH “Gerontologic Nursing+”) OR (MH “Health Services for the Aged”) or (“aging in place” or elders or elderly or geriatric* or gerontolog* or gerodontic* or old age or (seniors not “high school”) or “senior citizen*” or (older N3 (patient* or adult* or person* or people or man or men or woman or women)) or centenarian* or nonagenarian* or octogenarian* or septuagenarian* or sexagenarian* or dottering or decrepit or tottering or overaged or “oldest old”)
3. S1 and S2
4. (MH “Nursing Homes + ”)
5. (MH “Nursing Home Patients”)
6. (MH “Rehabilitation Centers”) OR (MH “Residential facilities”) OR (MH “Housing for the Elderly”)
7. ((“nursing home*” or “extended care*” or “care home*”)) OR (((convalescen* or senior* or “continuing care” or disabled or “old age” or geriatric* or “elder care*” or rehabilitat* or “long term care”) N2 (lodge* or facility* or home* or residence* or centre* or center*))) 70,416
8. S4 or S5 or S6 or S7
9. S3 or S8
10. (MH “Pain+”)
11. (pain* or discomfort* or soreness or ache* or aching or hurt*)
12. S10 or S11
13. (MH “Quality of Life”)
14. (“quality or life” or QoL or HRQoL)
15. “(“life satisfaction” or “life-satisfaction”)”
16. “(“wellbeing” or “well-being”)”
17. S13 or S14 or S15 or S16
18. S9 AND S12 AND S17

**Database**: SCOPUS < 1974 to August 4, 2022 > **(Total searches: 145)**

( ( TITLE-ABS-KEY ( elders OR elderly OR geriatric* OR old AND age OR senior* OR centenarian* OR nonagenarian* OR octogenarian* OR septuagenarian* OR sexagenarian* OR dottering OR decrepit OR tottering OR overaged OR “oldest old”)) OR ( TITLE-ABS-KEY ( older W/3 ( adult* OR person* OR people OR man OR men OR woman OR women)))) AND ( TITLE-ABS-KEY ( (“nursing home*” OR “extended care*” OR “care home*” OR ( ( senior* OR “continuing care” OR disabled OR “old age*” OR geriatric* OR “elder care*” OR rehabilitat* OR “long term care”) W/2 (lodge* OR facility* OR home* OR residence* OR centre* OR center*))))) AND ( TITLE-ABS-KEY (pain* OR discomfort* OR soreness OR ache* OR aching OR hurt*)) AND ( TITLE-ABS-KEY (“quality or life” OR qol OR hrqol OR hqol OR “life-satisfaction” OR “life satisfaction” OR “wellbeing” OR “well-being”)) AND

(LIMIT-TO ( DOCTYPE,“ar”) OR LIMIT-TO (DOCTYPE,“re”))

**Database**: Cochrane Library < 1993 to August 4, 2022 > **(Total searches: 341)**

1. [mh Geriatrics]
2. [mh Aged]
3. [mh “Health Services for the Aged”]
4. [mh “Senior Centers”]
5. (elders or elderly or geriatric* or old age or (seniors not “high school”)):ti,ab,kw
6. (older NEAR/3 (adult*or person* or people or man or men or woman or women) or centenarian* or nonagenarian* or octogenarian* or septuagenarian* or sexagenarian* or dottering or decrepit or tottering or overaged or “oldest old”):ti,ab,kw
7. #1 OR #2 OR #3 OR #4 OR #5 OR #6
8. [mh “Nursing Homes”]
9. [mh “Homes for the Aged”]
10. [mh “Rehabilitation Centers”]
11. [mh “Skilled Nursing Facilities”]
12. (nursing home* or extended care* or care home*):ti,ab,kw
13. ((senior* or continuing care or disabled or old age or geriatric* or elder care* or rehabilitat* or long term care) NEAR/2 (lodge* or facility* or home* or residence* or centre* or center*)):ti,ab,kw
14. #8 OR #9 OR #10 OR #11 OR #12 OR #13
15. [mh pain]
16. (pain* or discomfort* or soreness or ache* or aching or hurt*):ti,ab,kw
17. #15 OR #16
18. [mh ^“Quality of Life”]
19. (“quality or life” or QoL or HRQoL or HQoL):ti,ab,kw
20. ((life) NEAR/2 (satisfaction)):ti,ab,kw
21. (“wellbeing” or “well-being”):ti,ab,kw
22. {OR #18-#21}
23. #7 AND #14 AND #17 AND #22

**Database**: Proquest Dissertations and Theses global < 1853 to 2014 > **(Total searches: 52)**

1. noft(elders OR elderly OR geriatric* OR “old age*”)
2. noft(older NEAR/3 (adult* or person* or people or man or men or woman or women))
3. S1 OR S2
4. noft(“nursing home*” or “rehabilitation center*” or “skilled nursing facility*” or “care home*” or “extended care*”)
5. noft((senior* or “continuing care” or disabled or “old age” or geriatric* or (“elder care”) or rehabilitat* or “long term care”) NEAR/3 (lodge* or facility* or home* or residence* or centre* or center*))
6. S4 OR S5
7. noft(pain* or discomfort* or soreness or ache* or aching or hurt*)
8. noft(“quality of life” or QoL or HRQoL or HQoL or “life satisfaction” or “life-satisfaction” or “wellbeing” or “well-being”)
9. S3 AND S6 AND S7 AND S8

## Abbreviations

LTC: Long-term care
QoL: Quality of life
HRQoL: Health-Related Quality of Life
ADL: Activities of Daily Living
PRISMA-P: Preferred reporting items for systematic review and meta-analysis protocols
PROSPERO: International Prospective Register of Systematic Reviews
AMSTAR 2: Assessing the Methodological Quality of Systematic Reviews 2
QATQS: Quality Assessment Tool for Quantitative Studies
AXIS: Appraisal Tool for Cross-sectional Studies
CASP: Critical Appraisal Skills Program
MMAT: Mixed Methods Appraisal Tool
GRADE: Grading of Recommendation Assessment, Development and Evaluation guidelines
